# Antioxidant Supplementation for Management of Gestational Diabetes Mellitus in Pregnancy: A Systematic Review and Meta-Analysis of Randomised Controlled Trials

**DOI:** 10.1007/s13668-025-00636-1

**Published:** 2025-03-14

**Authors:** Paige van der Pligt, Glenn D. Wadley, I-Lynn Lee, Sara Ebrahimi, Sheree Spiteri, Kim Dennis, Shaun Mason

**Affiliations:** 1 Institute for Physical Activity and Nutrition (IPAN), School of Exercise and Nutrition Sciences, Deakin University, Geelong, 3220 Australia; 2 Department of Nutrition and Dietetics, Western Health, Footscray, Australia; 3 Department of Endocrinology, Sunshine Hospital, St Albans, Australia; 4 School of Exercise and Nutrition Sciences, Deakin University, Geelong, VIC 3220 Australia

**Keywords:** Gestational diabetes mellitus, Antioxidant, Supplementation, Pregnancy

## Abstract

**Purpose of Review:**

Gestational diabetes mellitus (GDM) is the most common medical complication of pregnancy globally. Hyperglycaemia and associated production of reactive oxygen species can lead to oxidative stress in pregnancy. However, the potential effectiveness of increased antioxidant intake in the management of GDM has not been widely examined. Its usefulness alongside medical nutrition therapy (MNT) for assisting glycaemic control in women with GDM is poorly understood. This review aimed to establish the effect of antioxidant supplementation on the risk and management of gestational diabetes mellitus (GDM).

**Recent Findings:**

A systematic review of intervention studies was conducted based on PRISMA guidelines. Databases searched were MEDLINE, CINAHL, Global Health, Scopus, Embase and Cochrane until September 2024. Random effects meta-analyses using Cochrane Review Manager software to establish the effect of antioxidant supplementation on glucose outcomes in women with GDM were conducted. A total of 13 studies (1380 participants) were included in the review with four different antioxidants used (selenium (*n* = 3); alpha-lipoic (*n* = 4); zinc (*n* = 5); e-3-gallate (*n* = 1)). Significant pre-post differences between antioxidant supplementation and control groups were found for fasting insulin (SMD, 95%CI) (-0.97 [-1.69 -0.24]; *p* = 0.009, HOMA-IR (-0.90 [-1.25, -0.54]; *p* < 0.0000, HOMA-B (-0.86 [-1.05, -0.67]; *p* < 0.00001 and QUICKI (1.09 [0.32,1.87]; *p* = 0.005 Heterogeneity was substantial (I^2^ > 50%, *p* < 0.05) for all models except for HOMA-B (I^2^ = 0%, *p* > 0.05).

**Summary:**

Antioxidant supplementation has possible benefit as an adjunct therapy to current dietary management for women with GDM. Further clinical trials are needed to establish the preferred type and dosage of antioxidants likely to be effective.

## Introduction

Gestational diabetes mellitus (GDM) is defined as any degree of glucose intolerance which is first recognised during pregnancy [1], and is the most common medical complication of pregnancy globally [2]. Prevalence of GDM has been estimated at approximately 15–20% [3, 4] although in some populations, prevalence may be as high as 25% [5]. Wide variation in diagnostic criteria and lack of adequate screening in some populations likely underestimate the number of pregnancies affected by GDM [5, 6]. Notwithstanding, rates of GDM are increasing rapidly and mirror those of obesity and type 2 diabetes mellitus (T2DM) [5].

A diagnosis of GDM increases risk for multiple short and long term maternal and fetal complications. Women who experience GDM are at higher risk for preeclampsia and caesarean section delivery [4], are more likely to develop GDM in subsequent pregnancies, and are at increased risk of both cardiovascular disease (CVD) and T2DM later in life [7]. Findings from a recent systematic review of 20 studies showed that women with GDM were ten-times more likely to develop future T2DM than women unaffected by GDM [8, 9]. Babies born to women with GDM are more likely to be born large for gestational age (LGA) and are at higher risk of neonatal hypoglycaemia, respiratory distress, shoulder dystocia and still birth [2, 5]. The offspring are further predisposed to developing future obesity and T2DM [5, 10]. Preventive approaches as well as strategies which focus on optimal management of GDM in the clinical setting are therefore critical to support best intergenerational health outcomes.

Medical Nutrition Therapy (MNT), together with self-monitoring of blood glucose levels are the first line treatments of GDM [11, 12]. The goal of MNT is to assist women to meet blood glucose and weight gain targets, in addition to providing a well-balanced diet to optimise both maternal and fetal health. Glycaemic control is primarily achieved by distributing a woman’s total carbohydrate intake evenly across the day (ranging from 36 to 65% total carbohydrate), and prioritising foods with a lower glycaemic index and higher fibre content [11]. Currently, recommendations emphasise that MNT should be individualised, according to comorbidities, learning and decisional capacity of the woman and cultural characteristics [12, 13]. With the predominant focus of MNT being on macronutrient intake, the vitamin and mineral intake recommendations do not differ from those specified to all pregnant women, irrespective of GDM status (e.g. folic acid, calcium, iron).

While a focus on adherence to dietary guidelines is fundamental for optimising maternal and neonatal health and growth, many women do not meet dietary guidelines during pregnancy [14–16] and overall diet quality is often sub-optimal throughout pregnancy [14]. Importantly, there may be an opportunity for assisting nutrient intake and glycaemic control in women diagnosed with GDM, which would involve appropriate nutritional supplementation alongside current MNT and dietary management of GDM. Moreover, there is an urgent need for management approaches which can be easily and effectively delivered in the clinical setting and might assist relieving increased pressures placed on clinicians and healthcare systems [17], that result from rapidly increasing rates of GDM.

It is well known that hyperglycaemia in diabetes increases the production of reactive oxygen species [18]. This results in increased oxidative stress and subsequent impairments in insulin-stimulated glucose uptake by peripheral tissues and insulin secretion by the pancreas [19–22] among multiple physiological alterations to cellular, enzyme and insulin-signalling pathways [19]. Previous research has found the presence of blood biomarkers of oxidative stress in women diagnosed with GDM [23] including markers of lipid peroxidation and DNA damage [23]. Antioxidant supplementation has been shown to reduce markers of oxidative stress in diabetes and recent data has shown significant reductions in fasting blood sugar and HbA1C% with antioxidant therapy in patients with T2DM [19, 20]. As such, antioxidant supplementation may be a promising therapeutic approach in managing T2DM [19]. Yet, the potential effectiveness of increased antioxidant intake in the management of GDM has not been widely examined, and its usefulness alongside MNT in clinical practice for assisting glycaemic control in women with GDM is not well understood.

To our knowledge, no systematic review has been previously published which reports the effectiveness of antioxidant supplementation on prevention and management of GDM. To better understand the effect of antioxidant intake as part of dietary management of GDM, the aim of this review was to systematically summarise glycaemic outcomes from intervention studies to date which have assessed the effect of antioxidant supplementation on risk and management of GDM.

## Materials and Methods

### Search Strategy and Selection Criteria

This systematic review was conducted based on the Preferred Reporting Items for Systematic Reviews and Meta-Analyses (PRISMA) criteria. A protocol was registered with PROSPERO, registration number CRD42022338311. MEDLINE, CINAHL, Global Health, Scopus, EMBASE and Cochrane databases were searched for the period up to September 2024 with no language or time restriction. Human intervention studies published in peer reviewed journals examining the effect of antioxidant supplementation on glycaemic outcomes relevant to GDM risk or management as the primary or secondary outcome were included as were studies which recruited women during pregnancy. Studies were excluded if they were not interventions, were animal studies or assessed the effect of dietary supplements not classed as antioxidants. Reference lists of all studies included in the review were screened to identify further studies for possible inclusion.

Key search words were gestational diabetes OR GDM OR Pregnancy-Induced Diabetes OR gestational diabetes mellitus OR hyperglycaemia in pregnancy OR gestational hyperglycaemia OR diabetes in pregnancy OR pregnancy complication OR pregnancy HbA1c OR pregnancy glycated haemoglobin OR pregnancy glycated haemoglobin OR pregnancy glucose AND intervention OR pilot OR randomized clinical trials OR RCT AND therapy OR supplement AND Antioxidant OR retinol OR vitamin a OR ascorbic acid OR vitamin c OR vitamin e OR alpha-tocopherol, OR carotenoid OR beta carotene OR lycopene OR oxy-carotenoids OR lutein OR zeaxanthin OR cryptoxanthin OR selenium OR glutathione peroxidase, superoxide dismutase OR catalase OR flavonoid OR lipoic acid OR co enzyme Q10 OR n-acetyl cysteine OR polyphenol OR zinc OR resveratrol OR green tea OR EGCG OR astaxanthin OR melatonin OR anthocyanin OR quercetin. Titles and abstracts were screened by two authors and duplicates were removed. Full text articles were screened based on the inclusion and exclusion criteria by four authors. Any conflict was resolved by discussion and articles were included by consensus.

### Data Extraction and Analysis

A data extraction template was developed to facilitate extraction of relevant information across study methodology and outcomes. Two authors (SE and SS) independently extracted the data addressing criteria consisting of study design and methodologies, intervention components and outcomes reporting the effect of antioxidant supplementation on glycaemic outcomes including haemoglobin A1c (HbA1c), fasting plasma glucose (FPG), fasting insulin, homeostatic model assessment for insulin resistance (HOMA-IR), homeostatic model assessment for beta cell function (HOMA-B) and quantitative insulin sensitivity check index (QUICKI). Any differences in the interpretation and extraction of data were resolved by discussion to minimise error.

### Risk of Bias Assessment

The Cochrane Risk of Bias tool was used to assess bias in the included studies [24]. The tool includes assessment of risk of bias across domains including the randomization process, assignment to the intervention, adherence to the intervention, missing outcome data, measurement of the outcome and selection of the reported result. Within each of the domains, assessment is made based on one or multiple items and a grading of ‘low risk’, ‘medium risk’ or ‘some concern’ is allocated for each domain. The final risk of bias rating is then given for each study based on the domain rating. The tool has been used previously for quality assessment reporting in similar studies and by members of the research team [20]. Two authors independently assessed risk of bias and any discrepancies were resolved by consensus.

### Reporting Bias Assessment

Small study effects were to be evaluated using funnel plots and Egger regression if at least 10 studies were included for an outcome [25]. However, all outcome comparisons consisted of less than 10 studies, and thus these evaluations were not conducted.

### Statistical Analysis

We conducted a meta-analysis for pre-post difference data when at least 2 study comparisons were available for a specific outcome. Where available, adjusted model data was preferentially used over raw data was used in the analysis. Mean changes were calculated from pre-treatment and post-treatment data if not directly provided. Standard deviations of pre-post changes were calculated assuming a correlation coefficient value of 0.7 if not directly provided. The value is based on imputations made using data from studies included the review [26–28] for which estimated coefficients ranged between 0.62 and 0.85 across different glucose outcomes [29]. We applied random effect models in analyses. The random effect model assumes that the effects being estimated across multiple studies are not identical but follow a distribution [30] and were therefore used due to evident heterogeneity in antioxidants investigated and study methodologies used, across included studies. Effect measures used were the standardized mean difference (SMD) and 95% CI. A p value of < 0.05 was considered statistically significant for all statistical tests. Heterogeneity between the included studies was evaluated using *I*^*2*^ and Cochran’s Q test, whereby *I*^*2*^ describes the percentage of variability that is due to overall heterogeneity [30, 31]. We used values of greater than 50% to define substantial heterogeneity alongside *p* < 0.05 [30, 31]. All statistical tests were conducted using Cochrane Review Manager (RevMan) software version 5.4 and data was used to generate forest plots with RevMan for each outcome.

## Results

### Search Results

**Fig. 1:**
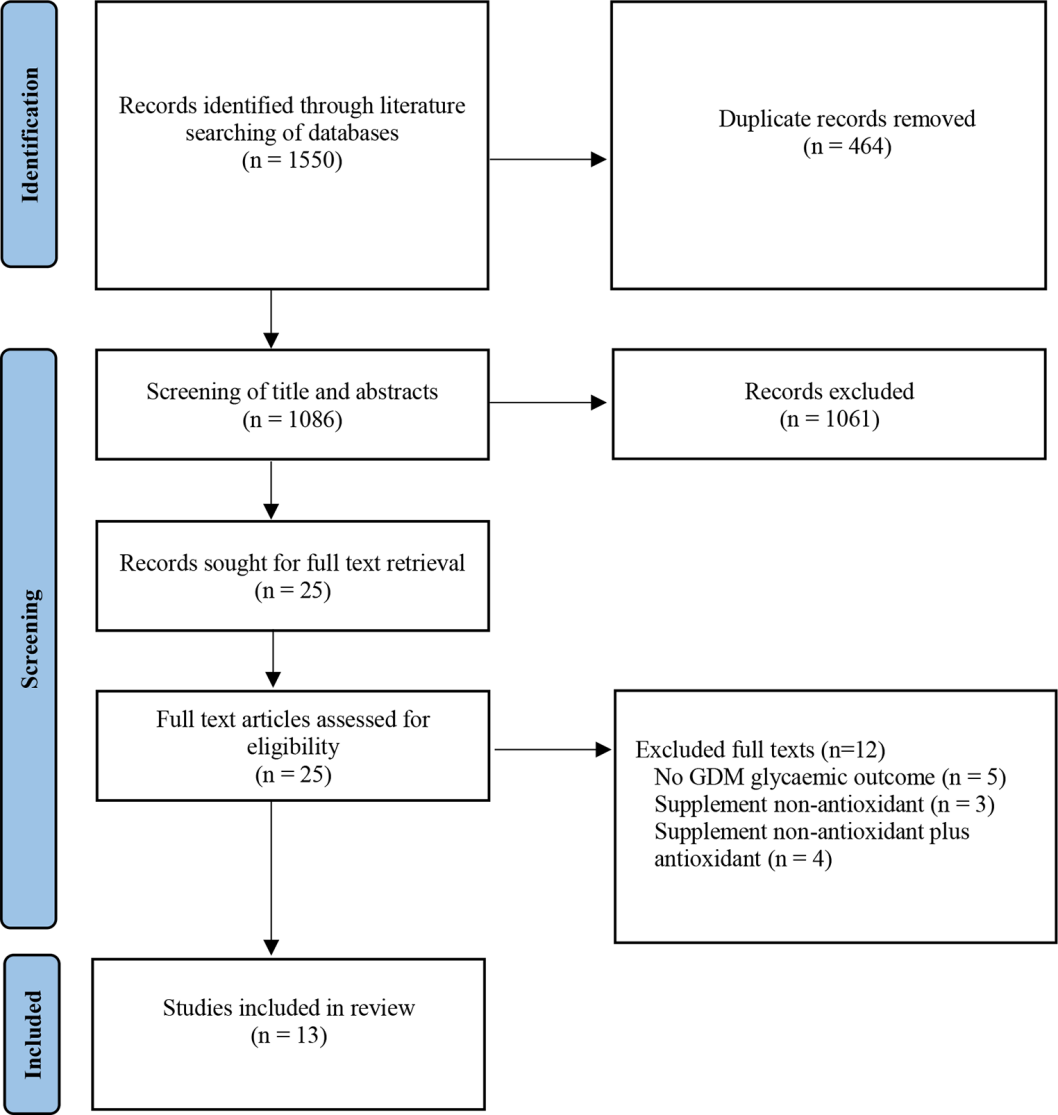
PRISMA flow diagram of included studies

Literature searching identified 1550 studies and after removal of 464 duplicates this left 1086 studies for title and abstract review. A total of 1061 studies did not meet the inclusion criteria, leaving 25 studies for full text review. Of these, 12 articles were excluded, and the remaining 13 studies were included in the review (Fig. 1). A summary of the included studies is presented in Table 1. Reasons for study exclusion were a lack of GDM glycaemic outcomes reported (*n* = 5), use of non-antioxidant supplements (*n* = 3), and concomitant intake of non-antioxidant supplements along with antioxidant supplements (*n* = 4).

**Table 1:** Summary of the included studies

Study and design	Participants	Setting and recruitment	Antioxidant and dosage	Intervention	Glycaemic measures	Glucose parameter outcomes
Aesmi et al.	Recruited: *n* = 70	Setting: maternity clinics	Selenium	(I) (*n* = 35): 200 µg selenium capsule daily for 6 weeks	FPG, serum insulin, HOMA-IR, HOMA-B, QUICKI	Change in FPG from baseline to intervention completion significantly different for the (I) group (-10.5 ± 11.9 mg/dL) compared to the (C) group (4.5 ± 12.9 mg/dL) p = < 0.001) and in serum insulin ((I) (-1.98 ± 11.25 µIU/mL (C) 5.26 ± 9.33 µIU/mL; *p* = 0.005), HOMA-IR ((I) (-0.84 ± 2.76 (C) 1.47 ± 2.46; p = < 0.001) and QUICKI score ((I) (0.008 ± 0.03, (C) -0.01 ± 0.01; *p* = 0.009); no significant difference in change for HOMA-B ((I) (-1.71 ± 43.62; (C) 16.30 ± 36.69; *p* = 0.06)
Iran	RR: not reported		200 µg/day			
2015 [26]	Retention: *n* = 65 (93%)	24–28 weeks gestation (mean 25.7 weeks)		(C) (*n* = 35): placebo capsule daily for 6 weeks		
			Compliance:			
Randomised double-blind placebo-controlled trial	Sample: GDM*, primigravida women with singleton pregnancies		100% reported for both groups	Diet and lifestyle: both groups maintained usual physical activity and dietary intake		
	Age: mean 28.6 years					
	BMI: mean 26.3 kg/m²		Safety: no reported side effects			
	Exclusion criteria^: women on insulin therapy or OHAs					
Aslfalah et al.	Recruited: *n* = 60	Setting: not reported	Alpha-lipoic acid (ALA) 100 mg/day	(I) (*n* = 30): 100 mg ALA capsule daily with lunch for 8 weeks	FPG	Significant decrease in FPG from baseline to intervention completion for both groups ((I) (101.43 ± 1.69 mg/dL, 83.56 ± 1.31 mg/dL; p = < 0.001); (C) (100.00 ± 1.24 mg/dL, 94.63 ± 1.18 mg/dL; *p* = 0.001))
Iran	RR: not reported					
2019 [40]	Retention: *n* = 60 (100%)	24–28 weeks gestation (mean 26.4 weeks)	Compliance: not assessed	(C) (*n* = 30): placebo capsule of cellulose acetate daily with lunch for 8 weeks		FPG significantly lower at intervention completion for the (I) group (83.56 ± 1.31 mg/dL) compared to the (C) group (94.63 ± 1.18 mg/dL) (p = < 0.001)
Randomised double-blind placebo-controlled trial	Sample: GDM*, pregnancy status not reported		Safety: no reported side effects	Diet and lifestyle: both groups followed GDM diet plan		
	Age: mean 31.1 years					
	BMI: mean 26 kg/m²					
	Exclusion criteria^: previous GDM, women on insulin therapy or OHAs; vitamin and mineral supplement use last six months					
Aslfalah et al.	Recruited: *n* = 70	Setting: not reported	Alpha-lipoic acid (ALA) 100 mg/day	(I) (*n* = 30): 100 mg ALA capsule daily for 8 weeks	FPG, HOMA-IR, QUICKI, insulin	Significant decrease in FPG from baseline to intervention completion for the (I) group (101.43 ± 1.69 mg/dL, 83.56 ± 1.31 [mean ± SEM] mg/dL (p = < 0.001) and the (C) group (100.00 ± 1.24 mg/dL, 94.63 ± 1.18 mg/dL (*p* = 0.001); no change in insulin ((I) 14.02 ± 0.47 mIU/L, 13.55 ± 0.59 mIU/L; *p* = 0.49); ((C) 13.37 ± 0.53 mIU/L, 13.96 ± 0.63 mIU/L; *p* = 0.58)
Iran	RR: not reported					
2019 [39]	Retention: *n* = 60 (85%)	24–28 weeks gestation (mean 26.4 weeks)	Compliance: not assessed Safety: no reported side effects	(C) (*n* = 30): 100 mg placebo capsule of cellulose acetate daily with lunch for 8 weeks		FPG significantly lower at intervention completion for the (I) group (83.56 ± 1.31 [mean ± SEM] mg/dL) compared to the (C) group (94.63 ± 1.18 mg/dL) (p = < 0.001) as was HOMA-IR (2.78 ± 0.13, 3.23 ± 0.13; *p* = 0.022); QUICKI was significantly higher for the (I) group (0.56 ± 0.01) compared to the (C) group (0.54 ± 0.005) (*p* = 0.019); no change in insulin ((I) (13.55 ± 0.59 mIU/L (C) 13.96 ± 0.63 mIU/L; *p* = 0.693)
	Sample: GDM*, pregnancy status not reported			Diet and lifestyle: both groups maintained usual physical activity and followed GDM diet plan		
	Age: mean 31.1 years					
	BMI: mean 26.8 kg/m²					
Randomised double-blind placebo-controlled trial						
	Exclusion criteria^: previous GDM, women on insulin therapy or OHAs; vitamin and mineral supplement use last six months					
Aslfalah et al.	Recruited: *n* = 70	Setting: hospital and diabetes clinic	Alpha-lipoic acid (ALA) 100 mg/day	(I) (*n* = 30): 100 mg ALA 100 mg capsule daily for 8 weeks	FPG and HbA1c	Significant change in FPG from baseline to intervention completion for both groups ((I) (101.43 ± 1.69 mg/dL, 83.56 ± 1.31 mg/dL (p = < 0.001); (C) (100.00 ± 1.24 mg/dL, 94.63 ± 1.18 mg/dL (*p* = 0.001)); no significant change in HbA1C for either group ((I) (5.29 ± 0.13%, 4.94 ± 0.13%; *p* = 0.059); (C) group (5.31 ± 0.12%, 5.09 ± 0.16%; *p* = 0.274))
Iran	RR: not reported					
2020 [38]	Retention: *n* = 60 (85%)	24–28 weeks gestation (mean 26.4 weeks)	Compliance: not assessed	(C) (*n* = 30): placebo capsule of cellulose acetate daily for 8 weeks		FPG significantly lower at intervention completion for the (I) group (83.56 ± 1.31 mg/dL) compared to the (C) group (94.63 ± 1.18 mg/dL) (p = < 0.001); no significant difference for HbA1c ((I) (4.94 ± 0.13%, (C) 5.09 ± 0.16%; *p* = 0.496))
	Sample: GDM*, pregnancy status not reported		Safety: no reported side effects	Diet and lifestyle: both groups maintained usual physical activity and followed the same diet; no further detail provided		
	Age: mean 31.1 years					
Randomised double-blind trial	BMI: mean 26.8 kg/m²					
	Exclusion criteria^: previous GDM, pregnancy, women on insulin therapy or OHAs; antioxidant					
	supplement use last six months					
Behrashi et al.	Recruited: *n* = 60	Setting: hospital and private clinics	Zinc Sulphate	(I) (*n* = 30): 25 mg zinc orally as zinc-containing syrup (5 ml of 5 mg/ml solution daily until term (38–40 weeks gestation)	Insulin dosage	Change in insulin dosage from baseline to intervention completion significantly different for the (I) group (8.76 ± 9.63 IU) compared to the (C) group (17.53 ± 10.54 IU); *p* = 0.001)
Iran	RR: not reported		25 mg/day			
2011 [35]	Retention: *n* = 60 (100%)	24–28 weeks gestation (mean not reported)		(c) (*n* = 30): 5 ml placebo oral syrup (with the same features without zinc) daily until term (38–40 weeks gestation)		Insulin dosage significantly lower at intervention completion for the (I) group (25.13 ± 10.78 IU) compared to the (C) group (32.76 ± 11.91 IU) (*p* = 0.012)
			Compliance: not assessed			
	Sample: GDM*, women with singleton pregnancies			Diet and lifestyle: not reported		
	Age: mean 28.5 years		Safety: no reported side effects			
	BMI: mean 26.4 kg/m²					
Randomised double-blind trial						
	Exclusion criteria^: previous GDM, women on insulin therapy					
Karamali et al.	Recruited: *n* = 50	Setting: maternity clinic	Zinc gluconate	(I) (*n* = 25): 233 mg zinc capsule daily for 6 weeks	Insulin requirements	Proportion of women requiring insulin at intervention completion not significantly different for the (I) group (0%) compared to the (C) group (4%) (*p* = 0.31)
Iran	RR: not reported		233 mg/day			
2016 [36]	Retention: *n* = 46 (92%)	24–28 weeks gestation (mean not reported)	(each containing 30 mg zinc)	(C) (*n* = 25): placebo capsule daily for 6 weeks		
	Sample: GDM*, pregnancy status not reported		Compliance: reported high for both groups	Diet and lifestyle: both groups maintained usual physical activity and dietary intake		
	Age: mean 29.6 years					
	BMI: mean 28.2 kg/m^2^		Safety: no reported side effects			
Randomised double-blind placebo-controlled trial						
	Exclusion criteria^: T1DM, T2DM, women on insulin therapy, zinc supplement use					
Karamali et al.	Recruited: *n* = 58	Setting: maternity clinic	Zinc gluconate	(I) (*n* = 29): 233 mg zinc capsule daily for 6 weeks	FPG, insulin, HOMA-IR, HOMA-B, QUICKI	Change in FPG from baseline to intervention completion significantly different for the (I) group (-6.6 ± 11.2 mg/dL) compared to the (C) group (0.6 ± 6.7 mg/dL) (*p* = 0.005) and in insulin ((I) (-1.3 ± 6.6µIU; (C) 6.6 ± 12.2µIU; *p* = 0.003), HOMA-IR ((I) (-0.5 ± 1.6; (C) 1.5 ± 2.7; *p* = 0.001), HOMA-B ((I) (-0.7 ± 25.0; (C) 26.5 ± 49.5; *p* = 0.01) and QUICKI ((I) (0.01 ± 0.01; (C) -0.01 ± 0.02; *p* = 0.004)
Iran	RR: not reported		233 mg/day			
2015 [27]	Retention: *n* = 53 (91%)	24–28 weeks gestation (mean not reported)	(each containing 30 mg zinc)	(C) (*n* = 29): placebo capsule daily for 6 weeks		
						FPG significantly lower at intervention completion for the (I) group (− 6.6 ± 11.2 mg/dl) compared to the (C) group (+ 0.6 ± 6.7 mg/dl) (*p* = 0.005) as was serum insulin ((I) (− 1.3 ± 6.6 µIU/mL; (C) + 6.6 ± 2.2 µIU/mL; *p* = 0.003), HOMA-IR ((I) (− 0.5 ± 1.6; (C) + 1.5 ± 2.7; *p* = 0.001) and HOMA-B ((I) (− 0.7 ± 25.0; (C) + 26.5 ± 49.5, *p* = 0.01); QUICKI significantly higher for the (I) group (+ 0.01 ± 0.01) compared to the (C) group (− 0.01 ± 0.02) *p* = 0.004)
Journal of Diab and its complications	Sample: GDM*, pregnancy status not reported		Compliance: reported high for both groups	Diet and lifestyle: both groups maintained usual physical activity and dietary intake		
	Age: mean 29.7 years					
	BMI: mean 28 kg/m^2^		Safety: no reported side effects			
	Exclusion criteria^: T1DM, T2DM, women on insulin therapy, zinc supplement use					
Randomised double-blind placebo-controlled trial						
Mandani et al.	Recruited: *n* = 60	Setting: not reported	Alpha-lipoic acid (ALA) 300 mg/day	(I) (*n* = 30): 300 mg ALA capsule daily for 8 weeks	FPG	Change in FPG from baseline to intervention completion significantly different for the (I) (-6.76 ± 0.97 [mean ± SEM] mg/dL group compared to the (C) group.(− 2.70 ± 0.65 mg/dL) (*p* = 0.001)
Iran	RR: not reported					
2021 [37]	Retention: *n* = 60 (100%)		Compliance: not assessed	(C) (*n* = 30): placebo capsule of cellulose acetate daily for 8 weeks		
		24–28 weeks gestation (mean 25.8 weeks)				
	Sample: GDM*, pregnancy status not reported		Safety: no reported side effects	Diet and lifestyle:		
	Age: mean 29.6 years			both groups followed the same diet plan; no further detail provided		
Randomised double-blind trial	BMI: mean 28.6 kg/m^2^					
	Exclusion criteria^: previous GDM, antioxidant supplement use last six months					
Najib et al.	Recruited: *n* = 60	Setting: outpatient hospital clinic	Selenium	(I) (*n* = 26): 100 µg selenium capsule daily for 12 weeks	FPG, 2HPPG, insulin, HbA1c, HOMA-IR	No significant difference in FPG change from baseline to intervention completion for the (I) group (3.51 ± 1.22 mg/dL) compared to the (C) group (-3.52 ± 3.53 mg/dL) (*p* = 0.49) or in 2HPPG ((I) (-12.96 ± 8.91 mg/dL; (C) -13 ± 7.54 mg/dL; *p* = 0.95), HbA1c ((I) (-0.21 ± 0.43; C -0.14 ± 0.25, *p* = 0.51), serum insulin ((I) (-1.13 ± 3.81 mIU/ml; C -0.01 ± 2.58 mIU/ml; *p* = 0.33) or HOMA-1R (I) (-0.45 ± 1.15; C -0.25 ± 1.13; *p* = 0.52)
Iran	RR: not reported		100 µg/day			
2019 [28]	Retention: *n* = 53 (88%)	24–28 weeks gestation (mean 27 weeks)		(C) (*n* = 28): placebo capsule daily for 12 weeks		FPG not significantly different at intervention completion for the (I) group (92.75 ± 9.21 mg/dL) compared to the (C) group (83.51 ± 2.12 mg/dL) (*p* = 0.25); no significant difference for 2HPPG ((I) (136.21 ± 13.97 mg/dL; C 137.42 ± 24.04 mg/dL; *p* = 0.87), HbA1c ((I) (5.35 ± 0.54; C 5.31 ± 0.55; *p* = 0.34), serum insulin (I) (15.96 ± 5.63 mIU/ml; C 15.13 ± 4.89 mIU/ml; *p* = 0.57) or HOMA-1R (I) (3.37 ± 1.27; C 3.04 ± 1.09; *p* = 0.31)
			Compliance: not assessed			
	Sample: GDM*, 65% multigravida, women with singleton pregnancies			Diet and lifestyle: both groups received advice from a nutritionist for anti-diabetic diets and light exercise		
	Age: mean 30.1 years					
	BMI: mean 28.3 kg/m²		Safety: no reported side effects			
Randomised double-blind placebo-controlled trial						
	Exclusion criteria^: women on insulin therapy or OHAs					
Ostadmohammadi et al.	Recruited: *n* = 65	Setting: not reported	Zinc gluconate	(I) (*n* = 27): 233 mg zinc gluconate plus 400-IU vitamin E capsule daily for 6 weeks	FPG, insulin, HOMA-IR, QUICKI	QUICKI significantly higher at intervention completion for the (I) group ((0.33 ± 0.02)) compared to the (C) group (0.32 ± 0.02) (*p* = 0.007); significantly lower insulin ((I) (11.4 ± 4.8 µIU/ml; C (4.7 ± 5.8 µIU/ml; *p* = 0.001) and HOMA-IR ((I) (2.5 ± 1.1; (C 3.4 ± 1.5; *p* = 0.002); no significant difference in FPG between the (I) group (89.3 ± 11.2 mg/dl) and the (C group (93.0 ± 10.7 mg/dl) (*p* = 0.22)
Iran	RR: not reported		233 mg/day plus	(C) (*n* = 27): placebo capsule of cellulose acetate daily for 6 weeks		
2019 [41]	Retention: *n* = 54 (83%)	24–28 weeks gestation (mean 25.5 weeks)	vitamin E			
			400IU/day	Diet and lifestyle: All participants instructed to consume a healthy diet and participated in a nutrition education class		
	Sample: GDM*, pregnancy status not reported					
	Age: mean 30.8 years		Compliance: not assessed			
	BMI: Not reported					
			Safety: no reported side effects			
Randomised double-blind placebo-controlled trial	Exclusion criteria^: women on insulin therapy; zinc and/or vitamin E supplement use last three months					
Roshanravan et al.	Recruited: *n* = 58	Setting: health centre	Zinc gluconate	(I) (*n* = 22): 30 mg zinc gluconate capsule daily between meals for 8 weeks	Vaspin, FPG, insulin	No significant correlation for the (I) group between Vaspin levels and FPG change (*r*=-0.38, *p* = 0.078) or insulin change (*r*=-0.002, *p* = 0.992) from baseline to intervention completion
Iran	RR: not reported		30 mg/day	(C) (*n* = 22): placebo capsule of cellulose acetate capsule daily between meals for 8 weeks		
2018 [42]	Retention: *n* = 44 (76%)	24–28 weeks gestation (mean not reported)				
			Compliance: not assessed	Diet and lifestyle: both groups received individual dietary education provided by a dietitian		
Randomised double-blind controlled trial	Sample: pregnant women with IGT*, pregnancy status not reported					
	Age: mean 29.7 years		Safety: no reported side effects			
	BMI: mean 27.6 kg/m^2^					
	Exclusion criteria^: T1DM, T2DM					
Yigit et al.	Recruitment: *n* = 227	Setting: university	Selenium	(I) (*n* = 112): 200 µg/day selenium capsule daily for 30 days (4 weeks) plus dietary regulation for GDM	FPG	Change in FPG significant for both the (I) group (-5.85 ± 8.53 mg/dL) and the (C) group (2.0 ± 3.01 mg/dL) from baseline to intervention completion and significantly higher for the (I) group (p = < 0.001).
Turkey	RR: not reported		200 µg/day			
2024 [33]	Retention: *n* = 227 (100%)	24–28 weeks gestation (mean not reported)				At intervention completion FPG significantly lower for the (I) group (82.14 ± 6.23 mg/dL) compared to the (C) group (86.14 ± 8.02) (p = < 0.001); a significantly higher proportion of women in the (I) group were found to have FPG values lower than the 1st -hour and 2nd -hour thresholds (< 140, < 120) compared to the C group (26% vs. 6%; 25% vs. 6% (p = < 0.001 for both)
Randomised controlled trial	Sample: GDM*, 73% multigravida, pregnancy status not reported		Compliance: not assessed			
	Age: mean 31.41 years			(C) (*n* = 115): dietary regulation for GDM without selenium supplementation		
	BMI: mean 27.5 kg/m^2^		Safety: no reported side effects			
				Diet and lifestyle: both groups received standard diet regulation provided by a dietitian		
	Exclusion criteria^: women on insulin therapy or OHAs; hormone therapy					
Zhang et al.	Recruited: *n* = 472	Setting: hospital	Epigallocatechin 3-gallate	(I) (*n* = 176): 500 mg Epigallocatechin 3-gallate capsule (EGCG) daily until full term	FPG, insulin, QUICKI, HOMA-IR, HOMA-B	Significant decrease in FPG from baseline to intervention completion in the (I) group (104.6 ± 8.7 mg/dl, 89.3 ± 6.5 mg/dl; *p* = 0.04) and in insulin (I) (15.7 ± 4.3 µIU/ml, 8.8 ± 4.9 µIU/ml; *p* = 0.03), HOMA-IR (3.8 ± 1.4, 2.0 ± 1.6; *p* = 0.02) and HOMA-B (56.5 ± 19.6, 45.4 ± 18.5; *p* = 0.01); QUICKI score significantly increased from baseline to intervention completion (0.45 ± 0.16, 0.62 ± 0.14; *p* = 0.03)
China	RR: not reported		500 mg/day	(C) (*n* = 150) 500 mg placebo capsule until full term		
2017 [32]	Retention: *n* = 226 (48%)	Beginning of the third trimester (29 weeks) (mean not reported)				FPG significantly lower at intervention completion for the (I) group (89.3 ± 6.5 mg/dl) compared to the (C) group (105.7 ± 6.4 mg/dl) (*p* = 0.02) as was insulin (I) (8.8 ± 4.9 µIU/ml; C) 16.7 ± 4.8 µIU/ml; *p* = 0.01), HOMA-IR ((I) (2.0 ± 1.6; C) 3.8 ± 1.6; *p* = 0.04) and HOMA-B (I) (58.3 ± 21.2; C) 45.4 ± 18.5; *p* = 0.02); QUICKI significantly higher for the I group (0.62 ± 0.14) compared to the (C) group (0.31 ± 0.18) (*p* = 0.03)
			Compliance: not assessed	Diet and lifestyle: both groups advised to not consume tea-containing products, followed prescribed GDM diet and recorded own dietary intake		
Randomised double-blind controlled trial	Sample: GDM*, women with singleton pregnancies					
	Age: mean 29.2 years		Safety: no reported side effects			
	BMI: mean 26.1 kg/m^2^					
	Exclusion criteria^: T1DM, T2DM, women on insulin therapy; habitual tea consumption					

*American Diabetes Association diagnostic criteria; (I) intervention group; (C) control group; ^complete list of exclusion criteria included in supplementary Table 1; (GDM) Gestational Diabetes Mellitus; (FPG) fasting plasma glucose; (QUICKI) quantitative insulin sensitivity check index; (HOMA-IR) homeostatic model assessment for insulin resistance; (HOMA-B) homeostatic model assessment for beta cell function; (T1DM) type 1 diabetes mellitus; (T2DM) type 2 diabetes mellitus (OHAs) oral hypoglycaemic agents; (BMI) body mass index, (HbA1C) haemoglobin A1C; (RR) response rate; Data presented as mean ± SD unless indicated otherwise

### Summary of Included Studies

Out of the thirteen studies, eleven were conducted in Iran, one in China [32] and one in Turkey [33]. All studies were clinical, randomised controlled trials and 12 were double blinded. All studies assessed the effect of antioxidant supplementation on management of GDM rather than risk of GDM and all used the American Diabetes Association diagnostic criteria for GDM [34] derived from a 75 g oral glucose tolerance test at 24–28 weeks gestation [34].

Mean age of women ranged from 28.5 [35] to 31.4 [33] years and mean BMI at recruitment was classified as overweight across all studies (mean 27.2 kg/m²). Recruitment ranged from 50 women [36] to 472 women [32] and the study which recruited 472 women had the lowest retention rate at 48% [32]. A 100% retention rate was observed for three out of the 13 studies [33, 35, 37]. Four out of the 13 studies reported recruiting women with singleton pregnancies only, as part of eligibility criteria [26, 28, 32, 35]. There was wide variation in the specific exclusion criteria which could impact on glucose parameter outcomes across studies. Exclusion criteria included previous GDM plus women on insulin therapy or oral hypoglycaemic agents (OHA) for four studies [33, 38–40], only women on insulin therapy or OHAs with no reference to previous GDM for two studies [26, 28], previous GDM and women on insulin therapy for one study [35], only women with previous GDM for one study [37], only women on insulin therapy for one study [41], T1DM or T2DM and insulin therapy for three studies [27, 32, 36] and only T1DM or T2DM for one study [42]. Intervention duration varied across studies from four weeks in one study [33], six weeks in four studies [26, 27, 36, 41] eight weeks in five studies [37–40, 42] and 12 weeks in one study [28], with two studies describing the intervention length as ‘until term’ [32, 35].

### Antioxidants and Effect on Glucose Parameters

Data summarising the within and between group intervention effects on glucose parameter outcomes are presented in Table 1. Three studies provided intervention group participants with selenium supplementation [26, 28, 33]. At a dosage of 200 µg/day for six weeks [26], significant and beneficial intervention effects on fasting glucose, serum insulin, HOMA-IR and QUICKI were shown for the intervention group compared to the control group. At the same dosage for 30 days (four weeks), significant and beneficial effects were shown for fasting glucose [33].

There was no significant intervention effect of selenium supplementation on glucose parameters, at a dosage of 100 µg/day for 12 weeks [28]. Three studies provided intervention group participants with alpha-lipoic acid (ALA) at a dosage of 100 mg/ day for eight weeks [38–40]. All three studies showed significant and beneficial effects of the intervention on fasting glucose with one study showing additional significant and beneficial effects on HOMA-IR and QUICKI [39]. One additional study provided women with ALA at a dosage of 300 mg/day for eight weeks [37] and showed significant and beneficial effects on fasting blood glucose.

A total of five studies provided the intervention group with zinc in the form of zinc sulphate (25 mg/day from 24 to 28 weeks gestation until term) [35], zinc gluconate (233 mg/day for six weeks) [27, 36], zinc gluconate at a lower dosage (30 mg/day for 8 weeks) [42] and a combined antioxidant supplement consisting of zinc gluconate (233 mg/day) plus vitamin E (400-IU) for six weeks [28]. There was a significant and beneficial effect of zinc supplementation on insulin dosage within and between groups [35] and on fasting blood glucose, insulin, HOMA-IR, HOMA-B and QUICKI [27]. A significant and beneficial intervention effect on insulin, HOMAR-IR and QUICKI was also observed when zinc was combined with vitamin E [41]. In the study which administered zinc gluconate at 30 mg/day for eight weeks, no significant effect was observed on fasting blood glucose or insulin [42] nor was there a significant effect on the proportion of participants requiring insulin in one of the studies which provided 233 mg/day for six weeks [36].

The one study conducted in China provided intervention group participants with 500 mg/day epigallocatechin 3-gallate from the beginning of the third trimester until term [32]. The intervention showed significant and beneficial within and between group effects on fasting plasma glucose, insulin, HOMA-IR, HOMA-B and QUICKI.

### Pre-Post Intervention Effect

**Fig. 2:**
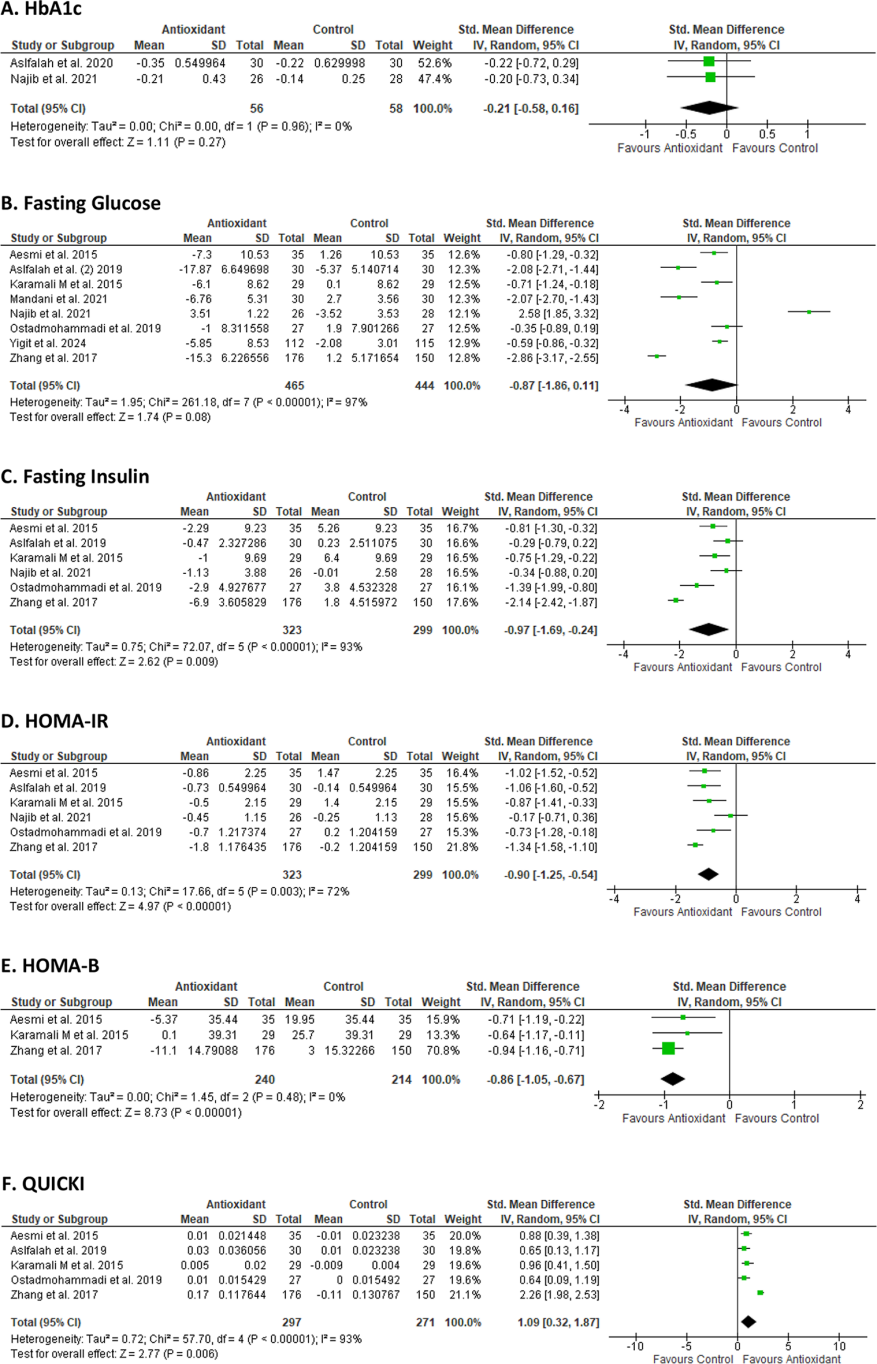
Forest plots summarising pre-post intervention effects

Meta-analysis data for six glucose parameter outcomes are presented in Fig. 2. Antioxidant supplementation had a significant effect on fasting insulin (SMD − 0.97 [-1.69 -0.24]; *p* = 0.009) (Fig. 2C), HOMA-IR (SMD − 0.90 [-1.25, -0.54]; *p* < 0.00001), (Fig. 2D) HOMA-B (SMD − 0.86 [-1.05, -0.67]; *p* < 0.00001) (Fig. 2E) and QUICKI (SMD 1.09 [0.32, 1.87]; *p* = 0.006) (Fig. 2F) when compared to control. There was no significant effect of antioxidant supplementation and HbA1C% (SMD − 0.21 [-0.58, 0.16]; *p* = 0.27) or fasting glucose (SMD − 0.91 [-2.13, 0.30], *p* = 0.14) from baseline to intervention completion for the intervention groups compared to the control groups. The meta-analyses showed substantial heterogeneity among the included studies which assessed fasting insulin (I^2^ = 93%, *p* < 0.00001), fasting glucose (I^2^ = 97%, *p* < 0.0001), HOMA-1R (I^2^ = 72%, *p* = 0.003) and QUICKI (I^2^ = 93%, *p* < 0.00001) but not for HOMA-B (I^2^ = 0%, *p* = 0.48) or HbA1C% (I^2^ = 0%, *p* = 0.96).

### Risk of Bias

**Fig. 3:**
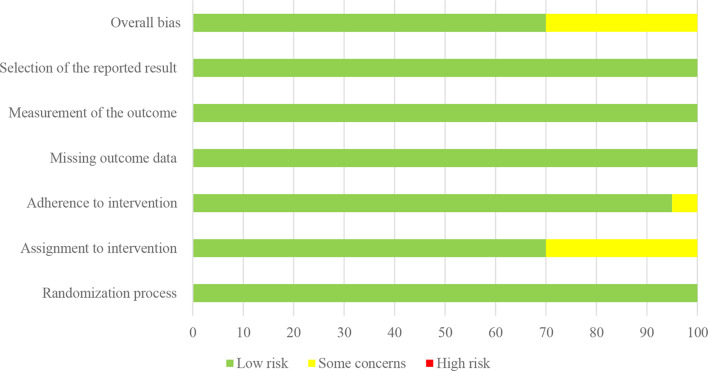
Risk of bias of included studies

A summary of the risk of bias assessment is presented in Fig. 3 with ratings for each domain and overall bias presented for all studies. We found an overall low risk of bias whereby nine studies were assessed as low risk [26, 27, 35–40]. The remaining four studies presented with ‘some concerns’ of bias [28, 33, 41, 42].

## Discussion

This systematic review was the first to summarise the available evidence assessing the use of antioxidant supplementation for management of GDM. The review has shown that antioxidant supplementation may be a potentially effective approach to assisting current MNT practice. This is best represented by findings of our review showing beneficial effects of antioxidant supplementation on fasting insulin, HOMA-IR, HOMA-B and QUICKI. Recognising the need for further clinical trials this review has contributed novel data regarding adjunct nutrition therapies which may benefit women with GDM and who cannot meet required antioxidant levels through dietary intake alone.

Increasing antioxidant intakes may be a useful approach in clinical practice for women diagnosed with GDM. GDM is associated with a heightened level of oxidative stress [43] due to the overproduction of reactive oxygen species and / or a defect in the antioxidant defence system [43]. Pancreatic ß cells are vulnerable to effects of oxidative stress which damages mitochondria, reduces insulin secretion and increases blood glucose [44, 45]. Adequate antioxidant intake might therefore be critical for blood glucose homeostasis [44]. With excessive oxidative stress leading to significant cellular damage through altering proteins, lipids and DNA [43], the body’s oxidative defence system during pregnancy is critical in protecting maternal health as well as promoting optimal fetal growth and development.

Our review included assessment of pregnant women and as such the included studies did not include studies assessing prevention of GDM in the preconception period. As the aetiology of GDM is complex and multifactorial [46], with genetic and environmental factors contributing to its development [46], is difficult to provide specific recommendations in current clinical practice for the prevention of GDM [47]. Despite the recent emergence of evidence to support pre and early pregnancy diet and physical activity modification as key lifestyle factors playing a beneficial role in reducing risk for GDM [48], some clarity is lacking regarding optimum intervention strategies required to achieve best health outcomes. However, as the review has shown favourable effects of antioxidant intake on glycaemic outcomes, there is a need to focus future work on the potential and crucial role of nutrition in GDM prevention in the preconception period and in early pregnancy.

Importantly, the role of diet in GDM management is pivotal and encouraging adequate intake of food groups in line with evidenced based MNT should not be displaced by reliance on nutrition supplementation alone. Whilst this review has shown that antioxidant supplementation may be a useful adjunct form of management alongside MNT, it could be possible that adequate levels of some of the antioxidants included in this review be reached by optimal dietary intake of key nutrients and food groups across pregnancy (e.g. fruits and vegetables, nuts, red meat, dairy, grains and seeds). However, studies in this review did not analyse antioxidant intake consumed by participants through the diet, and therefore it was not clear on the additional beneficial effect of supplementation in addition to dietary intake levels achieved on glycaemic outcomes. A threshold for beneficial effects on glycaemic outcomes is unknown until adequate evidence from robust clinical trials inform the most effective level of antioxidants required for intake to impact GDM outcomes.

Pregnancy is a period of vast lifestyle adaptation and a healthy dietary intake which adheres to guidelines can be challenging for many women [49]. For example, many women do not meet recommendations for fruit and vegetable intake during pregnancy [50–52]. For women diagnosed with GDM, early and pre pregnancy increased fruit and vegetable intake has been shown to be inversely associated with risk of developing GDM [53] and is associated with improved birth outcomes [54] compared to low intake of fruit and vegetables. Nutrition education and interventions which include evidenced based guidance on the importance of dietary antioxidant intake may be advantageous and strategies which focus on increased fruit and vegetable intake and other key food groups in pregnancy, for overall health benefits should continue to be prioritised. Education strategies in practice might include information related to the importance of antioxidant intake and potential benefits to glycaemic control, once further evidence is established. Notwithstanding, the results from this review reiterate the importance of dietary management for women diagnosed with GDM and the positive impact of dietary strategies in promoting beneficial glycaemic outcomes as well as the potential for future, further work which involves nutrition intervention and strategies.

In practice, financial costs associated with antioxidant supplementation use in pregnancy may be a barrier to intake, across different sociodemographic groups. Previous research has shown cost to be a barrier for compliance of nutritional supplements during pregnancy [55] and as such this needs to be a consideration in practice implementation. In terms of a healthcare systems approach, implementing antioxidant supplementation as part of routine clinical practice would require an economic analysis and consider the financial burden and resources required in GDM management including provision of insulin therapy, practitioner time and healthcare workforce support. Nevertheless, due to rapidly increasing rates of GDM globally and subsequent pressures placed on healthcare systems in managing women with GDM, it behoves us to consider opportunities that may present to enhance the care of women with GDM, achieve best patient outcomes whilst being practical and cost effective.

Despite the majority of included studies reporting favourable effects there was no statistical significance in the meta-analysis for fasting glucose with antioxidant supplementation. However, given the relatively small number of studies that were included, this warrants further research in the context of pharmacotherapy use during pregnancy based on fasting blood glucose levels. Assessment of pharmacotherapy use as an outcome in future research would be a useful addition to understanding impact of antioxidants on GDM management. Further, assessment of postprandial glucose levels as an outcome was not prioritised in studies in this review. Notwithstanding, in clinical practice, there is a need to utilise more technological advancements in glucose monitoring such as continuous glucose monitoring for outcome measures that would provide information regarding postprandial glucose and hyperglycaemia [56]. Utilising continuous glucose monitoring has the additional benefit of proving insight into the relationship between early pregnancy glycaemic patterns and GDM diagnosis [56] and could be highly beneficial in understanding the relationship between antioxidant intake and GDM management.

### Strengths and Limitations

There are both strengths and limitations to this review. Firstly, this review included RCTs with predominately a low risk of bias. Despite a small number of studies overall, we did not place time or language restrictions on searches and as such retrieved all available evidence to date. However, almost all studies included in this review were conducted in Iran. This reduces the ability to extrapolate findings to diverse, global populations. Further, we limited our search to recruitment of women during pregnancy which may have impacted the ability to retrieve studies that have targeted prevention of GDM by recruiting women in the preconception period.

Whilst we were not able to include all studies in the meta-analyses, those for which there were outcomes for at least two studies were included. We reported significant heterogeneity across studies on a number of outcome measures, yet we performed pre-post intervention effects and random effects models to account for heterogeneity which was a strength of this review. We were unable to conduct sensitivity analyses and subgroup analyses due to the small number of studies included, and therefore, important aspects such as dose-related effects were unable to be evaluated.

## Conclusion

This review has offered novel and important insight into the potential role of antioxidant supplementation as an adjunct therapy to dietary management of GDM in the clinical setting. It has further reiterated the importance of nutrition management in promoting beneficial glycaemic outcomes for women with GDM. Further RCTs will be useful to address current knowledge gaps in considering potential future use of antioxidant supplementation alongside MNT for women diagnosed with GDM.

## Key References

- S. Mason, L. Parker, P. van der Pligt and G. Wadley. “Vitamin C supplementation for diabetes management: A comprehensive narrative review”. Free Radical Biology and Medicine, volume 194, pp. 255–283, January 2023. 10.1016/j.freeradbiomed.2022.12.003.

This comprehensive review outlines the physiological effects of vitamin C on diabetes outcomes and describes the utility of antioxidants on a range of diabetes measures. Although further RCTs are required, vitamin c may be a useful adjunct therapy in various patient groups with diabetes. This reference is ‘of importance’.

- H. Zakaria, S. Abusanana, B. Mussa, A. Dhaheri, L. Stojanovska, M. Mohamad, S. Saleh, H. Ali and L. Ismail. “The Role of Lifestyle Interventions in the Prevention and Treatment of Gestational Diabetes Mellitus”. Medicina, volume 59(2), pp. 287, January 2023. 10.3390/medicina59020287.

This review summarises evidence assessing efficacy of lifestyle treatments for prevention and management of GDM. It reiterates the need for personalised approaches to diet and physical activity interventions in pregnancy and the vast capacity for nutrition to have positive effects on GDM outcomes. Further, individualised approaches are required. This reference is of ‘outstanding importance’.

- D. Shrivastav, P. Dabla, J. Sharma, A. Viswas and R. Mir. “Insights on antioxidant therapeutic strategies in type 2 diabetes mellitus: A narrative review of randomized control trials”. World Journal of Diabetes, volume 14(6), pp. 919–929, June 2023. 10.4239/wjd.v14.i6.919.

This comprehensive review evaluated the effect of antioxidant therapy on glycamic outcomes and antioxidant atatus in patients with type 2 diabetes mellitus. It showed positive outcomes and utility for including antioxidant therapy in patients with type 2 diabetes mellitus. The pathway for the potential effectiveness between oxidative stress in diabetes and antioxidant usage is outlined. This reference is of ‘outstanding importance’.

## Data Availability

No datasets were generated or analysed during the current study.
